# Serum Metabolic Profiling Analysis of Gout Patients Treated with Traditional Chinese Medicine Tongfengtai Granules Based on Gas Chromatography-Mass Spectrometry

**DOI:** 10.1155/2020/7404983

**Published:** 2020-04-26

**Authors:** Jianyong Zhang, Hong Pan, Jingjing Xie, Jing Wang, Ruyi Wang, Xia Qiu, Li Zhong, Tengyan Li, Yuya Xiao, Min Xiao, Yanying Zhang, Ertao Jia, Yubao Jiang, Binbin Wang

**Affiliations:** ^1^Department of Rheumatism, Shenzhen Traditional Chinese Medicine Hospital, Shenzhen, Guangdong, China; ^2^The Fourth Clinical Medical College of Guangzhou University of Chinese Medicine, Shenzhen, Guangdong, China; ^3^Graduate School, Peking Union Medical College, Beijing, China; ^4^Center for Genetics, National Research Institute for Family Planning, Beijing, China; ^5^The Department of Medical Genetics and Developmental Biology, School of Basic Medical Sciences, Capital Medical University, Beijing, China

## Abstract

Gout has become a public health problem that seriously threatens human health. Traditional Chinese medicines (TCMs) have a long history of treating gout and have some advantages compared with the conventional medicines. Compound TCM Tongfengtai granules are gradually being used for clinical treatment of gout, but its mechanism is still unclear. The purpose of this study was to explore the metabolic profiling of serum from gout patients before and after treatment with Tongfengtai granules and identify the differential metabolites and related metabolic pathways. A total of 40 gout patients hospitalized in Shenzhen Traditional Chinese Medicine Hospital from 2018 to March 2019 were recruited in the current study, and serum samples from these patients before and after treatment with Tongfengtai granules were collected. Gas chromatography-mass spectrometry (GC-MS) assay was used to identify serum metabolites. The OPLS-DA VIP method was used to screen for potential metabolic biomarkers, and MetaboAnalyst 4.0 was used to identify related metabolic pathways. The result showed that there was a significant difference in the concentrations of six metabolites in the serum after treatment: D-galactose, lactic acid, 3-hydroxybutyric acid, D-pyran (type) glucose, alanine, and L-isoleucine. Except D-pyran (type) glucose, the serum concentrations of the other five metabolites were all significantly reduced. Besides, pathway enrichment analysis found that these potential metabolic biomarkers were mainly involved in lactose degradation and the glucose-alanine cycle. Thus, the serum metabolic profiling of gout patients treated with Tongfengtai granules changed, and the differential metabolites and related metabolic pathways might provide clues for understanding the mechanism of Tongfengtai granules.

## 1. Introduction

Gout is an inflammatory disease caused by the deposition of monosodium urate crystals (MSUC) in synovial fluid or other connective tissue, the main cause of which is a disorder of purine metabolism and/or a decrease in uric acid excretion [1]. The most typical clinical manifestations of this disease are hyperuricemia and gouty acute arthritis caused by MSUC deposition. Some patients with persistent and untreated gout have symptoms such as tophi and joint limitation, swelling, and deformity [2]. The reported prevalence of gout varies from country to country, but the disease affects 0.1%–10% of the population around the world [3]. Recent studies have suggested that the prevalence of gout is increasing in both developed and developing countries, and the patients are tending to be younger such that the disease has become a serious public health problem [4–6].

At present, the most commonly used drugs for the treatment of gout are nonsteroidal anti-inflammatory drugs (NSAIDs), corticosteroids, colchicine, allopurinol, febuxostat, and probenecid, all of which act by reducing pain and swelling or the production of uric acid [7]. Because each drug has its own side-effects and limitations, the choice of medication to be used should always be made carefully. Traditional Chinese medicines (TCMs) have been used for a long time for the treatment of gout and have some advantages over these conventional medicines. Numerous clinical trials of TCMs have now been carried out, and they are widely used for the treatment of gout in clinical practice [8, 9].

Tongfengtai granules is a TCM that is composed of a mixture of seven Chinese herbal medicines, including shancigu (*Iphigenia* bulb), tufuling (rhizoma *Smilacis glabrae*), qinjiao (radix *Gentianae macrophyllae*), bixie (*Dioscoreae Hypoglaucae* rhizoma), chishao (radix *Paeoniae rubra*), shanzhuyu (*Cornus officinalis*), and chuanniuxi (radix *Cyathulae officinalis* Kuan). It is currently used for the treatment of gouty nephropathy and rheumatism, which are characterized by “damp heat” and “kidney deficiency”. The major functions of Tongfengtai granules are to clear heat and eliminate dampness, to detoxify and reduce turbidity, to relieve pain, and to protect the kidneys. They are suitable for patients who cannot use hormones, for those who have digestive tract diseases, or liver or kidney dysfunction, and for those who cannot use nonsteroidal or other anti-inflammatory drugs over the long term.

Although our previous clinical and animal studies have demonstrated that Tongfengtai granules have beneficial effects on gout, the mechanisms involved remain to be determined. Several previous studies have shown that mass spectrometry-based metabolomics is a powerful tool for the interrogation of the mechanisms of action of TCMs in the treatment of diseases [10, 11]. Gas chromatography-mass spectrometry (GC-MS) is one of the key analytical tools used in metabolomics, and it has been widely used in TCM studies. Serum is a readily accessible and informative biofluid, and serum metabolite profiles help our understanding of the mechanisms of TCM action [12]. In the present study, we recruited 40 hospitalized gout patients and used a GC-MS-based metabolomic approach and multivariate statistical methods to analyze the serum metabolites of gout patients before and after treatment with Tongfengtai granules.

## 2. Materials and Methods

### 2.1. Reagents

Methanol and acetonitrile were purchased from Adamas Reagent Co. Ltd (Shanghai, China), N-methyl-N-(trimethylsilyl) trifluoroacetamide (MSTFA) (98%) from Shanghai Bailingwei Technology Co., Ltd (Shanghai, China), pyridine (99.0%) from TCI-Tixi Ai (Shanghai) Chemical Industry Development Co., Ltd (Shanghai, China), and methoxyamine hydrochloride (98%) and heptadecanoic acid (98%) from Shanghai Maclean Biochemical Technology Co., Ltd (Shanghai, China).

### 2.2. Clinical Samples and Serum Collection

Forty gout patients who were hospitalized at Shenzhen Traditional Chinese Medicine Hospital between 2018 and March 2019 were included in this study, all of whom were 20–70 years old. Thirty of these patients were dampness-heat syndrome, accounting for 75% of all cases. Diagnoses of gout were made according to the criteria proposed by the American College of Rheumatology in 1977. The patients also met the diagnostic criteria for gout published in the 1999 “Diagnostic Efficacy Standard for TCM Syndrome”. Blood samples were collected from the patients before and after treatment with Tongfengtai granules for ∼1 week; the average action time was 7.5 days, the shortest was 6 days, and the longest was 10 days. Then, the blood samples were immediately centrifuged at 1,000 g for 10 min at 4°C, and the serums were separated and stored at −80°C until analysis.

The Tongfengtai granules were composed of 10 g of *Iphigenia* bulb, 45 g of rhizoma *Smilacis Glabrae*, 15 g of radix *Gentianae macrophyllae*, 30 g of *Dioscoreae Hypoglaucae* rhizoma, 10 g of radix *Paeoniae Rubra*, 6 g of *Cornus officinalis*, and 10 g of radix *Cyathulae officinalis* Kuan. If the patient had “Cold-Dampness Syndrome”, 15 g of turmeric, 15 g of garter snake, 15 g of common monkshood mother root, and 6 g of scorpion were added. If the patient had “Phlegm-Stasis Syndrome,” 10 g of *Pinellia* tuber, 10 g of white mustard seed, 10 g of Thunberg fritillary bulb, 10 g of peach seed, and 10 g of safflower were added. Finally, if the patient had “Pi-Deficiency Syndrome,” 20 g of largehead Atractylodes rhizome and 20 g of coix seed were added.

### 2.3. GC-MS Analysis

#### 2.3.1. Sample Preparation

The serum samples collected before and after treatment were numbered 1–80. After thawing at room temperature, 100 *μ*L of each were pipetted into 1.5 mL Eppendorf tubes, 50 *μ*L of heptadecyl carbonate (1 mg/mL) were added as an internal standard, and 200 μL of ice-cold acetonitrile were also added, and then the tubes were vortexed for 30 s and ultrasonicated for 5 min in a low-temperature environment. The mixtures were then centrifuged at 12,000 rpm for 10 min at 4°C, and the supernatants were transferred to gas chromatography vials and dried under nitrogen at a low temperature; then, 30 *μ*L of methoxyamine pyridine solution (15 mg/mL) was added. The mixtures were then incubated at 70°C for 1 h, and after cooling to room temperature, 30 *μ*L of MSTFA derivatization reagent was added, and silanization was carried out at 70°C for 1 h. After cooling, 20 *μ*L of each reaction mixture was loaded into cannulae and subjected to GC-MS to determine their chemical compositions. Further 20 *μ*L of each serum sample were placed into 2°mL Eppendorf tubes as quality control (QC) samples and treated in the same way.

#### 2.3.2. GC-MS Assay

GC-MS was performed using an AI1310-Trace1300-TSQ8000Evo (ThermoFisher Technology Co., Ltd.). The GC conditions were inlet temperature 280°C; split mode 20 : 1; temperature programme: initial temperature 70°C, held for 5 min, then warming to 300°C at 8°C/min, and then held for 3 min. The MS detection conditions were transmission line temperature 280°C; ion source temperature 250°C; solvent delay 6 min; and acquisition scan range (*m*/*z*) 40–600 Da. The injection volume was 1 *μ*L. The injection sequences were QC1, QC2, QC3, sample numbers 1–10; QC4, sample numbers 11–20; QC5, sample numbers 21–30; QC6, sample numbers 31–40; QC7, sample numbers 41–50; QC8, sample numbers 51–60; QC9, sample numbers 61–70; and QC10, sample numbers 71–80.

#### 2.3.3. Data Processing and Analysis

First, background subtraction was performed on the raw GC-MS data using Thermo Xcalibur data processing software (ThermoFisher Technology Co., Ltd.). Then, the National Institute of Standards and Technology (NIST) database was used to perform qualitative analysis of the mass spectrum peaks corresponding to the chromatographic peak signals at different retention times. Third, the chromatographic peak area of each substance was compared with the peak area of the internal standard substance (heptadecanoic acid) to calculate the relative content of each substance in different samples. Finally, the relatively quantitative data were stored in Excel file format and imported into the SIMCA-P14.1 software package (Umetrics, Umeat, Sweden). Following this, the outputs were subjected to principal component analysis (PCA) and orthogonal projections to latent structures discriminant analysis (OPLS-DA). In addition, considering that the retention times and chromatographic peaks of each substance in the standards and samples can be matched (data not shown), we did not perform experiments to calculate the retention index (RI), which is a limitation.

Variable importance in projection (VIP) values were used to screen the metabolites for an effect of treatment. The VIP value is the most commonly used method for the evaluation of data generated using the OPLS-DA supervised analysis method. In general, the larger the VIP value is, the greater the contribution of the variable to differences between the two groups. In this experiment, VIP values >1.5 were accepted, indicating that a metabolite was present at significantly different serum concentrations before and after treatment.

#### 2.3.4. Pathway Enrichment Analysis

The online metabolic data analysis platform MetaboAnalyst 4.0 (https://www.metaboanalyst.ca/) was used to identify the metabolic pathways containing metabolites that were present at differing concentrations [13]. The data were processed using the “Enrichment Analysis” model.

## 3. Results

### 3.1. Serum Metabolic Profile

**Figure 1:**
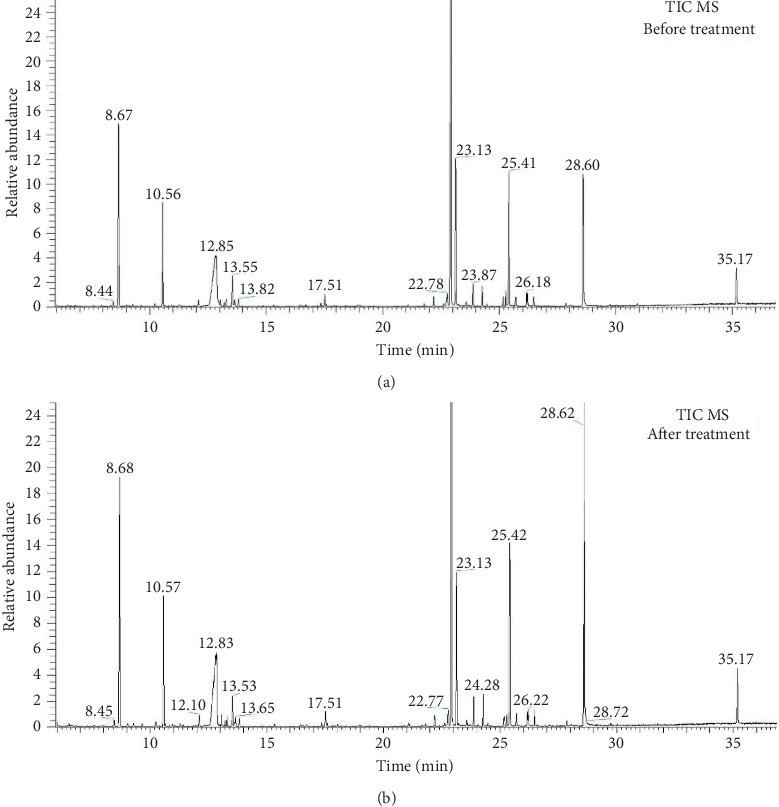
Typical serum chromatogram (a) before treatment and (b) after treatment.

Typical serum chromatograms for a gout patient before and after Tongfengtai granules treatment are shown in Figures 1(a) and 1(b). These show changes in the concentrations of metabolites before and after treatment. We normalized the integral data for the each metabolite to obtain relatively quantitative data.

### 3.2. OPLS-DA Analysis of Serum Metabolic Profiles

**Figure 2:**
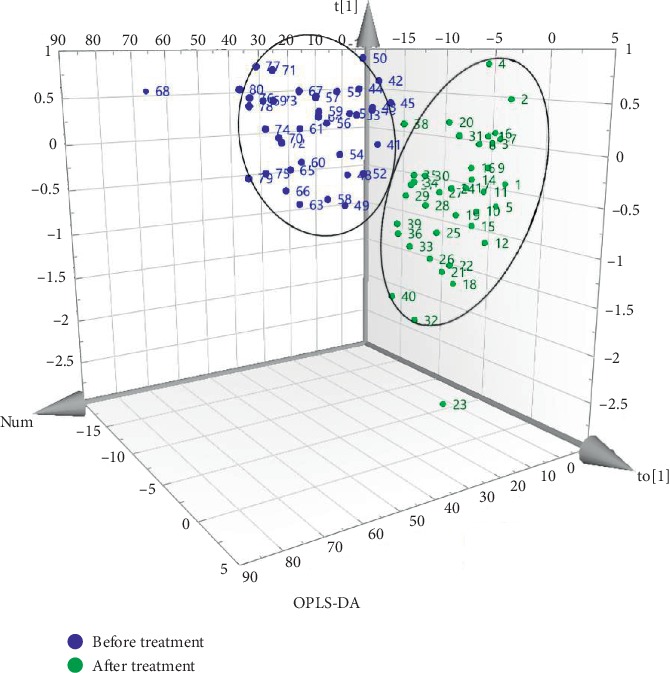
Clustering of OPLS-DA model scores before and after treatment.

To further characterize the metabolites that were affected by treatment with Tongfengtai granules, a supervised OPLS-DA pattern recognition method was used to remodel the serum metabolic profiles of gout patients before and after treatment. As shown in Figure 2, there were significant differences in serum metabolites before and after treatment in the supervised mode.

### 3.3. Screening for Serum Metabolites Affected by Tongfengtai Granule Treatment

After establishing the OPLS-DA model, a VIP value of >1.5 was chosen for the selection of candidate metabolites. In this way, six metabolites that differentiated the serum metabolic profiles before and after treatment were identified: D-galactose, lactic acid, 3-hydroxybutyric acid, alanine and L-isoleucine (concentration reduced by treatment), and D-pyran (type) glucose (concentration increased by treatment) (Table 1).

### 3.4. Enrichment Analysis of the Corresponding Metabolic Pathways

**Figure 3:**
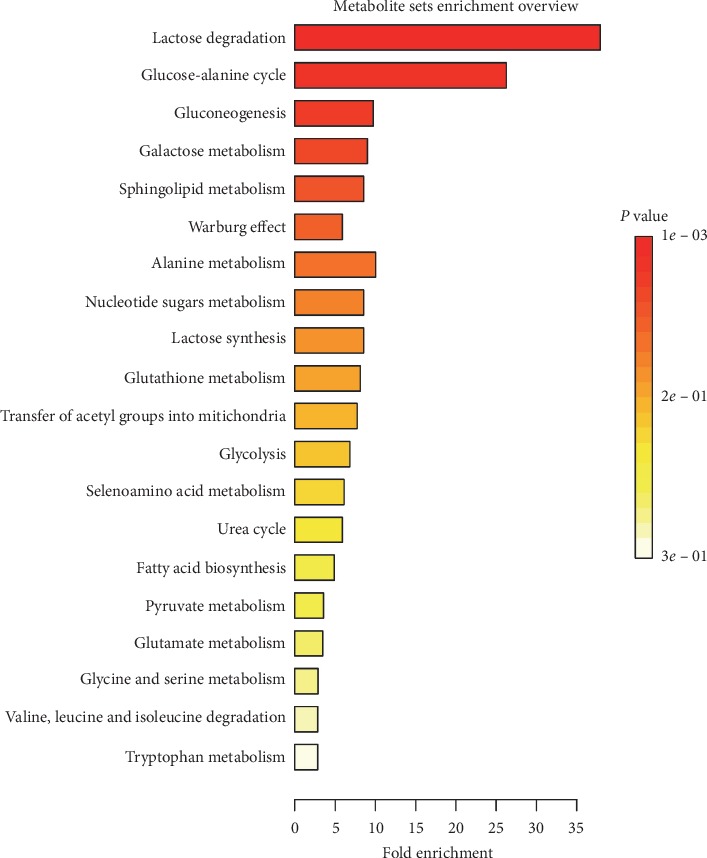
The metabolic pathways potentially affected by treatment.

To determine the metabolic pathways that might be affected by treatment, MetaboAnalyst 4.0 was used to perform pathway enrichment analysis using the identified metabolites. The Small Molecule Pathway Database (SMPDB) was used as the reference database: this consists of 99 metabolite groups that comprise normal human metabolic pathways. The metabolic pathways implicated are shown in Figure 3. Two principal metabolic pathways were implicated: lactose degradation and the glucose-alanine cycle.

## 4. Discussion

In the present study, GC-MS metabolomic analysis was used to identify serum metabolites that were affected by the treatment of gout with Tongfengtai granules. The OPLS-DA VIP approach was employed to screen potential metabolic biomarkers, and MetaboAnalyst 4.0 was used to identify the related metabolic pathways. This analysis revealed differences in the concentrations of six serum metabolites before and after treatment with Tongfengtai granules: D-galactose, lactic acid, 3-hydroxybutyric acid, D-pyran (type) glucose, alanine, and L-isoleucine. With the exception of D-pyran (type) glucose, these metabolites demonstrated significantly lower serum concentrations. These potential metabolic biomarkers were found to be primarily involved in lactose degradation and the glucose-alanine cycle.

A number of studies have shown that hyperuricemia and gout cause significant changes in the concentrations of specific metabolites. In a recent prospective cohort study, higher plasma concentrations of cysteine, glutamine, phenylalanine, threonine, and long-chain acylcarnitine were found to be associated with a higher risk of developing hyperuricemia over 6 years in a middle-aged population without hyperuricemia [14]. Although the metabolic markers identified may differ because of differences in the materials and methods used in each study, metabolomics research is very useful to improve understanding of the pathophysiology of gout and to identify novel therapies [15, 16].

Treatment of gout with TCMs may reverse the pathological process by affecting the concentrations of these metabolic biomarkers [17–20]. In a recent study of the effects of Quzhuotongbi decoction in hyperuricemic rats, GC-MS analysis showed that the concentrations of uric acid, lactic acid, pyruvic acid, and ornithine were significantly higher, and the degree of jaundice and the concentrations of uronic acid, amino acids (aspartate, proline, glutamine, serine, pyroglutamate, and glutamate), and glucose were significantly lower [17]. The changes in the concentrations of lactic acid and glucose in the present study were consistent with this report, with Tongfengtai granule treatment being able to normalize the concentrations of these metabolites.

A poor diet or endogenous defects that increase uric acid production or inhibit uric acid excretion can cause an increase in uric acid concentration and increase the risk of gout [21]. The kidney is principally responsible for the excretion of uric acid, which is the end-product of purine catabolism in humans. However, when uric acid is not excreted efficiently, its serum concentration increases, which can lead to gout [22]. Monocarboxylic organic acids, such as oxalic acid, lactic acid, and ketone bodies (acetoacetic acid and *β*-hydroxybutyric acid), are metabolized by the same pathways as uric acid; therefore, abnormal concentrations of these metabolites can also result in a hyperuricemia and gout [23]. Lactose degradation involves the hydrolysis of *α*-lactose by lactase to form D-glucose and D-galactose, which are used as a source of energy [24]. Furthermore, the product of anaerobic metabolism of glucose or lactose is lactic acid. The glucose-alanine cycle assists with the maintenance of glycemia and prevents an increase in lactic acid as a consequence of higher pyruvate concentration [25].

## 5. Conclusions

In summary, the present study has shown that Tongfengtai granules may be capable of reversing the pathophysiology of gout by affecting the concentrations of specific metabolites and the related metabolic pathways. However, the effects of these metabolic changes on the pathogenesis of the disease and the mechanisms whereby TCMs have beneficial effects on gout remain to be determined.

## Figures and Tables

**Table 1:** Serum metabolites present at different concentrations before and after treatment in the metabolomic screen.

Metabolite	VIP	Trend
D-Galactose	2.56	Reduce
Lactic acid	2.23	Reduce
3-Hydroxybutyric acid	1.85	Reduce
D-Pyran (type) glucose	1.79	Increase
Alanine	1.60	Reduce
L-Isoleucine	1.50	Reduce

## Data Availability

The data used to support the findings of this study are available from the corresponding author upon request.
